# High Count Rate Electron Probe Microanalysis

**DOI:** 10.6028/jres.107.043

**Published:** 2002-12-01

**Authors:** Joseph D. Geller, Charles Herrington

**Affiliations:** Geller MicroAnalytical Laboratory, Topsfield, MA 01983

**Keywords:** electron probe microanalysis, EPMA, gas flow proportional detector, GFPC, pulse pile-up rejection, sealed proportional counter, x ray, XePC, x-ray detectors

## Abstract

Reducing the measurement uncertainty of quantitative analyses made using electron probe microanalyzers (EPMA) requires a careful study of the individual uncertainties from each definable step of the measurement. Those steps include measuring the incident electron beam current and voltage, knowing the angle between the electron beam and the sample (takeoff angle), collecting the emitted x rays from the sample, comparing the emitted x-ray flux to known standards (to determine the *k*-ratio) and transformation of the *k*-ratio to concentration using algorithms which includes, as a minimum, the atomic number, absorption, and fluorescence corrections.

This paper discusses the collection and counting of the emitted x rays, which are diffracted into the gas flow or sealed proportional x-ray detectors. The representation of the uncertainty in the number of collected x rays collected reduces as the number of counts increase. The uncertainty of the collected signal is fully described by Poisson statistics. Increasing the number of x rays collected involves either counting longer or at a higher counting rate. Counting longer means the analysis time increases and may become excessive to get to the desired uncertainty. Instrument drift also becomes an issue. Counting at higher rates has its limitations, which are a function of the detector physics and the detecting electronics.

Since the beginning of EPMA analysis, analog electronics have been used to amplify and discriminate the x-ray induced ionizations within the proportional counter. This paper will discuss the use of digital electronics for this purpose. These electronics are similar to that used for energy dispersive analysis of x rays with either Si(Li) or Ge(Li) detectors except that the shaping time constants are much smaller.

## 1. Uncertainty Measurements in EPMA

The use of the term “accuracy” has been defined by the International Standardization Organization (ISO) and American National Standards Institute [1] as a qualitative term to describe the closeness between the measured and true value. As such, numbers should not be used to describe accuracy. To describe this quantitatively the term “uncertainty” is used [2]. The uncertainty of an experiment is a result of the individual uncertainties of each part of the experiment that may add error to the result. Normally, the individual uncertainties are summed in quadrature, where the result is equal to the square root of the sum of the squares. The overall uncertainty is referred to as the uncertainty budget.

A partial list of the uncertainties include what errors will result in measuring the incident electron beam current (is the meter linear and how is it measured?), the electron beam voltage, knowing the angle between the electron beam and the sample (takeoff angle), collecting the emitted x rays from the sample, comparing the emitted x-ray flux to known standards (to determine the *k*-ratio) and transformation of the *k*-ratio to concentration using algorithms which includes, as a minimum, the atomic number, absorption, and fluorescence corrections.

**Fig. 1:**
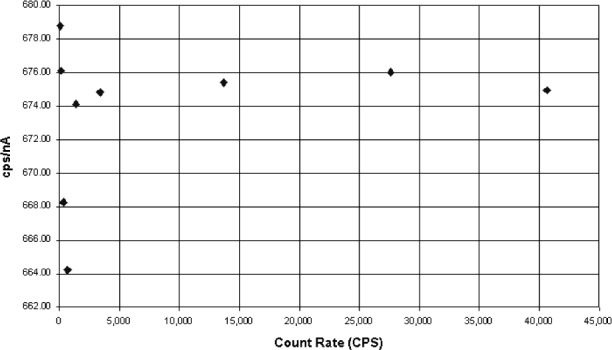
Large uncertainties at low count rates. Is it a non-linearity or from poor counting statistics?

An illustration of the count rate effect is seen in Fig. 1 where the copper Kα count per unit beam current is plotted against the count rate per second. It appears there is a non-linearity at low count rate. This deviation is simply a matter of not having enough counts. To get a 1 % uncertainty (2σ) at least 40 000 counts are required in the integral. When longer integration times are used the low count rate numbers fall in line with the rest.

It is the analyst’s responsibility to define the magnitude of the uncertainty for each step of the process. This process is qualitatively and quantitatively difficult. Adding to the difficulty is the idea that the uncertainties may change for different types of samples, the standards used, the counting statistics, and the correction factors used to finally determine the concentrations. For instance, if the sample analyzed is a stoichiometric compound or a homogenous alloy, a traceable calibration standard may be available which is of the same composition as the unknown sample. The uncertainty impact is insignificant and the correction algorithms should cancel (the correction factors will then be unity). However, if no standards are available one must rely on the correction algorithms. The resulting uncertainty will be difficult to determine. One must develop a strategy to do this. It may involve using pure elements, or end member oxides as standards to determine the composition of a sample similar to the unknown. The resulting uncertainty may be used in the budget.

Independent of the uncertainties described above, improving the statistics in the detected signal will always help to reduce the uncertainty in this part of the measurement, thus reducing the overall budget. However, if the uncertainty budget is large without considering the counting statistics, there may be no significant advantage to counting longer or at higher rates. For instance, if the relative uncertainty of the measurement of some element in an alloy is 10 %, improving the counting statistics from 2 % to 1 % while incurring a four times (from 5 min to 20 min) increase in the counting time may not be justified.

What is involved in improving the counting statistics? The counting time and rate have already been mentioned. Unfortunately, the count rate cannot be infinitely increased. Besides the limitations of increased sample heating, large focused electron beams from higher beam currents, and beam current stability, there are processes within the proportional counter and detector electronics limiting the count rate. To understand these issues we must understand how the x-ray proportional counter works.

## 2. The X-Ray Proportional Counter

**Fig. 2:**
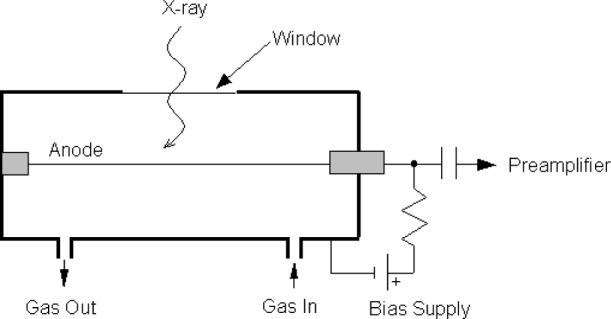
Side window gas flow proportional counter system.

In the EPMA, the incident electron beam interacts with the sample producing characteristic x rays. Part of the emitted x-ray flux undergoes Bragg diffraction and those x rays of the proper wavelength are focused to a line, which is occupied by the entrance of a proportional counter. The x rays must first be transmitted through a window before it gets into the gaseous environment of the counter. Figure 2 shows a typical side window type proportional counter used in JEOL^1^,^2^ EPMAs. The window materials used in current instruments are mesh supported stretched propropylene, mylar or beryllium. Polypropylene is used for the soft x rays, from atomic number four (Be) and higher. The mylar window is generally used for “K” line elements on the PET and LIF crystals. The Be window is generally used on a sealed proportional counter since the shorter wavelength x rays have near 100 % transmission for the elements diffracted by LiF crystals.

The most commonly used proportional counter gases are either P-10 (90 % Ar with 10 % methane) or xenon and a quench gas, such as methane or carbon dioxide. The stretched polypropylene window is considered to be semi-permeable to vacuum. This is why the P-10 gas is connected to a gas supply. The flow rate is very low. The pressure inside the counter is normally 760 torr. For the sealed counter the xenon gas may be at a partial pressure of 1300 Pa to 7800 Pa (10 torr to 60 torr). The partial pressure is selected to have the incident x rays stop near the counter wire, preventing the higher energy x rays from hitting the back wall of the counter. In the middle of the counter there is an anode wire (this may be a gold coated tungsten wire) of 25 μm, or so, in diameter. Once the x rays enter the counter how are they detected?

## 3. X-Ray Detection

When the x-ray enters the counter it ionizes the counter gas generating free electron-ion pairs. References [3–5] are excellent sources for the counter physics. The counter gas loses an electron in the process leaving the gas positively charged. A photoelectron is produced which has an energy equal to the energy of the incident x ray less the energy required to produce an electron-ion pair. The ionization potential of the argon is 15.17 eV and 12.1 eV for xenon. However, we must consider that more than one electron can be knocked out from the outer ring of the counter gas atom. The effective ionization potential is the average energy required to produce the electron-ion pair and is 26.4 eV and 20.8 eV, respectively, for Ar and Xe.

The x-ray counter is considered a proportional counter, if operated in the correct voltage range. The proper voltage (about 2000 V) is obtained when the magnitude of the number of electron pairs is proportional to the x-ray energy of the incoming photon (just like a Si(Li) detector). For a CuKα x ray (which has a wavelength of 1.54 Å or energy of 8040 eV) in xenon gas the number of ionizations is 8040 eV/20.8 or 386. For TiKα (which has an energy of about 4510 eV) there would be about half the counter pulse amplitude, or 217 ion pairs. If the counter voltage is too high there will be an uncontrolled avalanching effect where the incoming x ray will cause the entire counter gas to be ionized. This is the way a Geiger counter works. If the potential is too low the positive ions and the free electrons will recombine before they reach the anode. At higher voltages the counter acts like a ionization chamber with no secondary electrons occurring and the gas amplification is the same for all incoming photons.

The proportional counter can be used like a Si(Li) detector, except that the resolution is very poor in comparison. One must consider, however, that the x-ray photons incident on the counter are monochromatic, after being diffracted by the crystal. Typical FWHM (full width at half maximum intensity) resolution numbers for MnKα are 130 eV for Si(Li) and about 18 % of the x-ray photon energy, or 1061 eV for the proportional counter. Obviously, this counter is of not much use for analysis without a diffracting crystal in front of it. However, with EPMA it is possible to get (*n*) multiple order reflections at the same crystal position. Consider Bragg’s Law, where *n*λ = 2*d* sin*θ*, λ is the x-ray wavelength, and 2*d* the crystal lattice spacing in Å, and *θ* the angle the crystal makes with the sample. It is possible to have x-ray peaks at the same spectrometer crystal position (sin*θ*), one being a first order reflection and another being a second or third order reflection at two or three times the energy. In this case the counter energy resolution is adequate to separate the reflection orders.

Quenching in the counter limits the duration of the discharge in the counter. This is important for high counting rates. The quench gas in a flow counter is continuously replenished, while in a sealed counter it is slowly used up, limiting the number of counts to about 10^9^. The quench gas works in several ways to shorten the counter discharge. One way is that they neutralize some of the positive counter-gas ions by electron donation. Both the positive ions and the pre-amplifier resistor help to reduce the effective potential preventing avalanche. However, since the mobility of the positive ions is about 1000 times slower than the faster moving electrons, the ions form a space-charge shield around the counter wire. This serves to reduce the counter voltage. We see this as a pulse shrinkage when going to higher count rates. This is a fundamental limitation for high count rate detection of x rays that can fluoresce the counter gas.

**Fig. 3:**
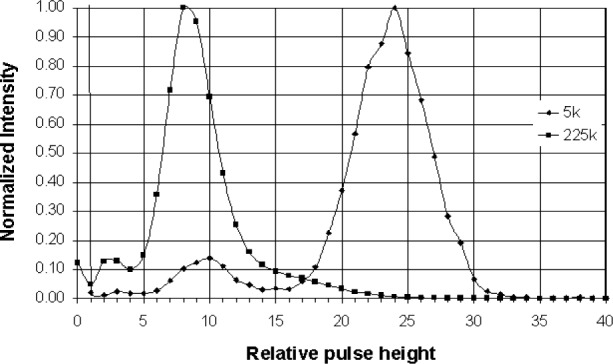
Pulse height distribution for Ti*k*α. At high count rates there is pulse shrinkage.

**Fig. 4:**
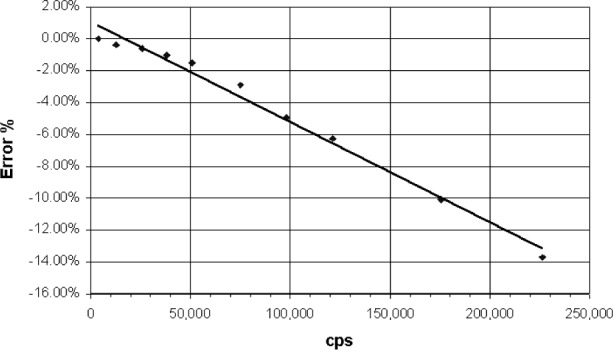
Ti*k*α count rate losses at high rates due to pulse shrinkage and escape peak loss.

**Fig. 5:**
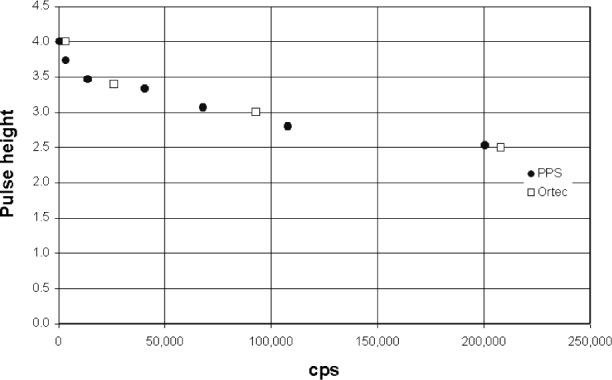
Pulse shrinkage for CuKα with count rate.

**Fig. 6:**
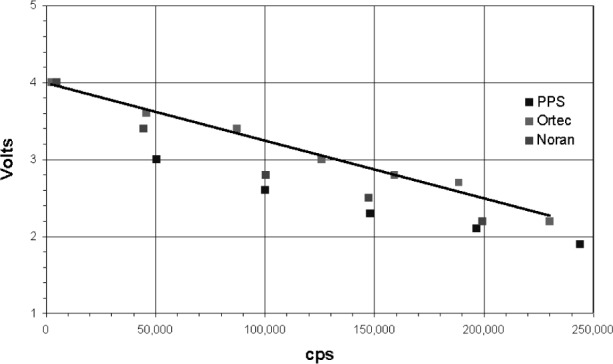
Pulse shrinkage for AlKα with count rate.

The absorption edge energy of Ar is 3.2 keV, while that for Xe is about 4.782 keV. When an incoming x ray exceeds this energy the detector gas atoms are ionized and a ArKα x ray can be generated. An escape peak is seen if this x ray leaves the counter. The effective energy left in the counter is the incoming energy less the absorption edge energy of the fluoresced counter gas. This gives a double peak in the pulse height distribution (Fig. 3). At higher count rates the ion cloud around the counter wire reduces the effective counter voltage (and gas gain), which causes the pulse height distribution to shift to lower voltages (Fig. 3). If the escape peak shifts to zero amplitude those pulses will be lost. Figure 4 illustrates the effect for TiKα where there is a linear loss with count rate. At 50 000 cps the loss is 2 %. To prevent this loss a “window” can be placed around the more intense peak at the pulse amplitude of the incoming photon, excluding the escape peak. At high count rates the window must follow the peak as the pulse shrinks to prevent count losses. The illustration in Fig. 5 shows the pulse shrinkage for CuKα using two different amplification systems. The PPS^3^ (for a description see Sec. 5) and Ortec^4^ are seen to have similar shrinkage with count rate. For AlKα the pulse shrinkage is plotted against count rate for the PPS, Ortec and Noran^5^ systems (Fig. 6). This illustrates all three systems have similar pulse shrinkage.

## 4. Counter Dead Time

The dead time is the time after an x ray is detected that no ionizations occur if another x ray enters the counter. The dead time is related to the time that the positive ion cloud around the wire decays. The dead time is thought [3] to be about 0.2 μs, however a longer time is required for the counter to produce a full amplitude pulse. Dead time varies linearly with pulse height. Counters also suffer from coincidence losses where two x rays enter the counter within the resolving time of the electronics where one pulse of twice the amplitude is generated. The equation used for this calculation relates the true counting rate, *I*_true_ to the observed *I*_obs_ and time losses, *τ* (in microseconds), of the detection system.

$$I_{\text{true}}=I_{\text{obs}}/\left(1-I_{\text{obs}}\;\tau \right).$$ (1)

**Fig. 7:**
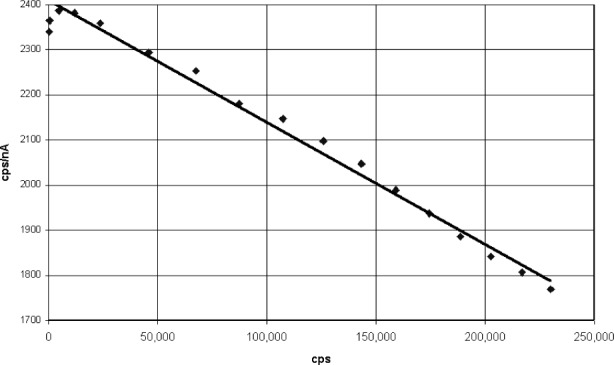
AlKα dead time characteristics for Ortec electronics.

**Fig. 8:**
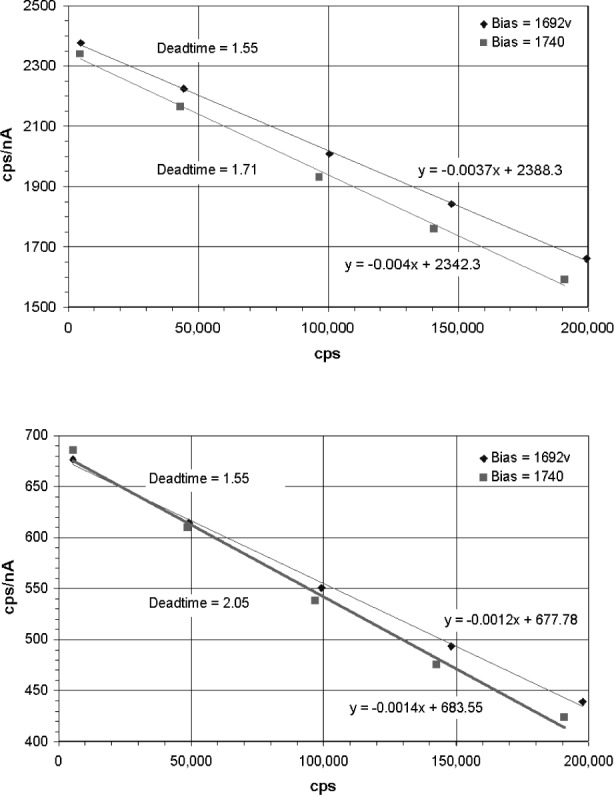
a. AlKα dead time characteristics for Noran PCS electronics. Notice the dead time changes with counter voltage. b. CuKα dead time characteristics for Noran PCS electronics. Notice the dead time changes with counter voltage.

Dead time is often measured by plotting the count rate as a function of count rate per incident beam current. The slope of the line is divided by the *y* axis zero intercept to obtain the dead time. This is illustrated in Figs. 7, 8a, and 8b, respectively, for AlKα and CuKα. We carefully measured the dead times at different amplifier gains and counter voltages and found them to be constant for the Ortec and PPS systems. With the Noran we found the dead times varied as a function of counter voltage. We do not propose an answer for this phenomenon but caution the analyst to use the proper dead times when making measurements.

**Fig. 9:**
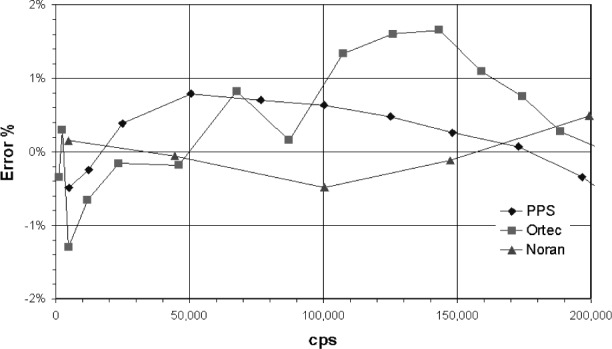
Count rate performance for dead-time and pulse shifted AlKα x rays. The Noran and PPS systems are within 0.5 % error.

**Fig. 10:**
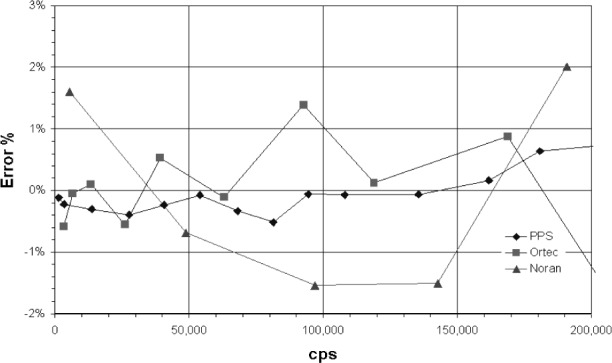
Count rate performance for dead-time and pulse shifted CuKα x rays. The PPS system is within 0.5 % error.

When the proper dead times are used for the Ortec and PAC (the dead time is automatically correction for the PPS system since it has pulse pile up rejection) it is feasible to count up to 200 000 cps. Figures 9 and 10 illustrate this. The lines connecting the points are only for help in visualizing the data. The PPS system is seen to have about 50 % of the error compared to Ortec and the PAC systems. For copper the PPS system has a counting rate error within 0.5 % to 150 000 cps.

## 5. Counting Electronics

Most EPMAs have preamplifiers, high voltage supplies, amplifiers, single channel analyzers and counters. The purpose of the preamplifier is to amplify the counter ionizations. It is usually one or two stages of amplification and an attenuating resistor. The signal is ac coupled to the high voltage supply. The amplifier further amplifies the signal for the single channel analyzer where the operator can select the appropriate voltage levels of the amplified pulses to count.

The past 20 years have seen the counting electronics go from manually operated to computer controlled systems. We have recently introduced more modern pulse processing electronics, similar to those used in Si(Li) detectors for the past many years. The improvements consist of adding pulse pile-up restoration to prevent multiple x rays from being amplified and shaped within the time resolving capabilities of the electronics. When a pulse is being processed and another pulse arrives before the process is completed, both pulses are rejected and a correction is made to the live counting time. In this way the dead time is corrected digitally instead of using Eq. (1). In a single electronics module, the pulse processing system (PPS) is interfaced to a computer through a high speed serial line. It contains a high voltage supply, preamplifier, amplifier, single channel analyzer and 32 bit counter.

With advances in electronics it is possible to improve the count rate uncertainty for EPMA analyses. Without extending the analysis time, counting rates up to 200 000/s can be used. The observed uncertainty can be reduced to 0.5 %.

## Footnotes

1 Japan Electron Optical Laboratory, Akishima, Japan.

2 NIST disclaimer: Certain commercial equipment, instruments, or materials are identified in this paper to foster understanding. Such identification does not imply recommendation or endorsement by the National Institute of Standards and Technology, nor does it imply that the materials or equipment identified are necessarily the best available for the purpose.

3 PPS self contained pulse processing system from Geller MicroAnalytical Laboratory, Topsfield, Ma.

4 Ortec model 142 preamplifier, 456 high voltage and 490 amplifier/single channel analyzer. Oak Ridge, Tennessee.

5 Noran “PCS” pulse counting system, from ThermoNoran, Middleton, Wisconsin.
